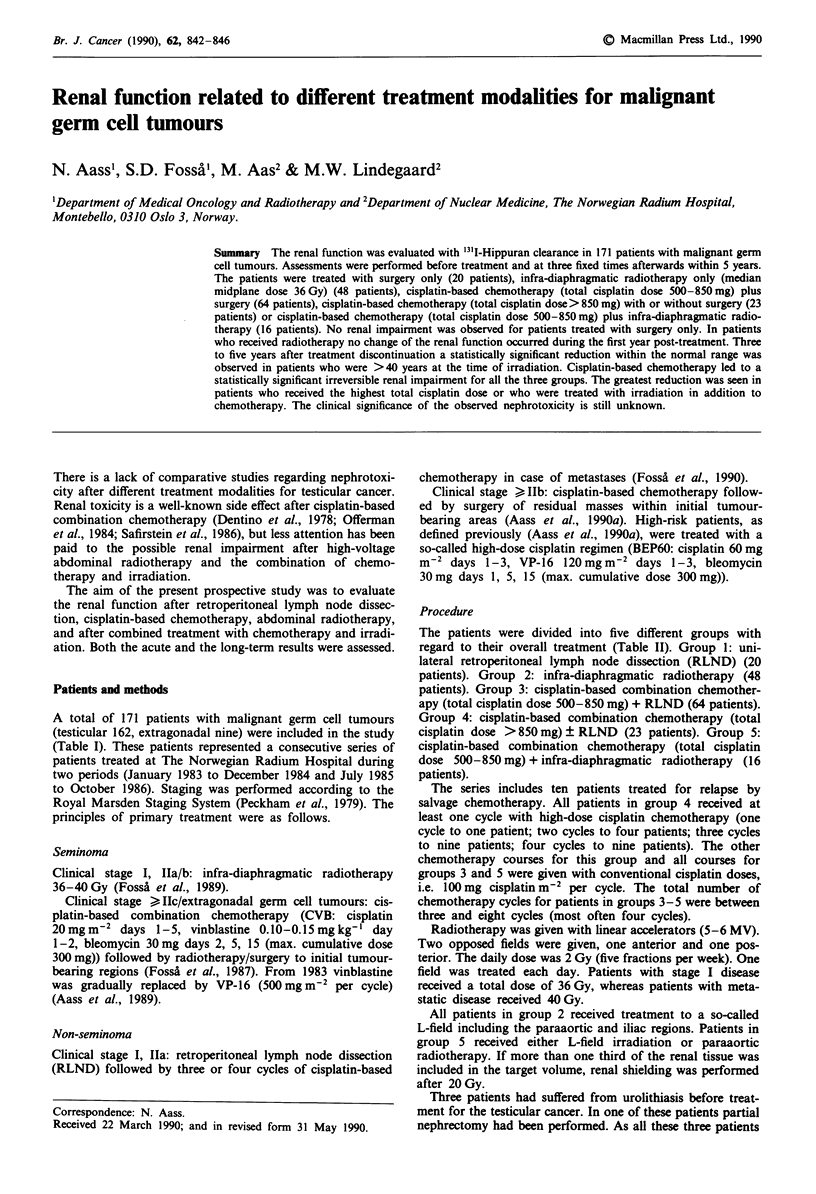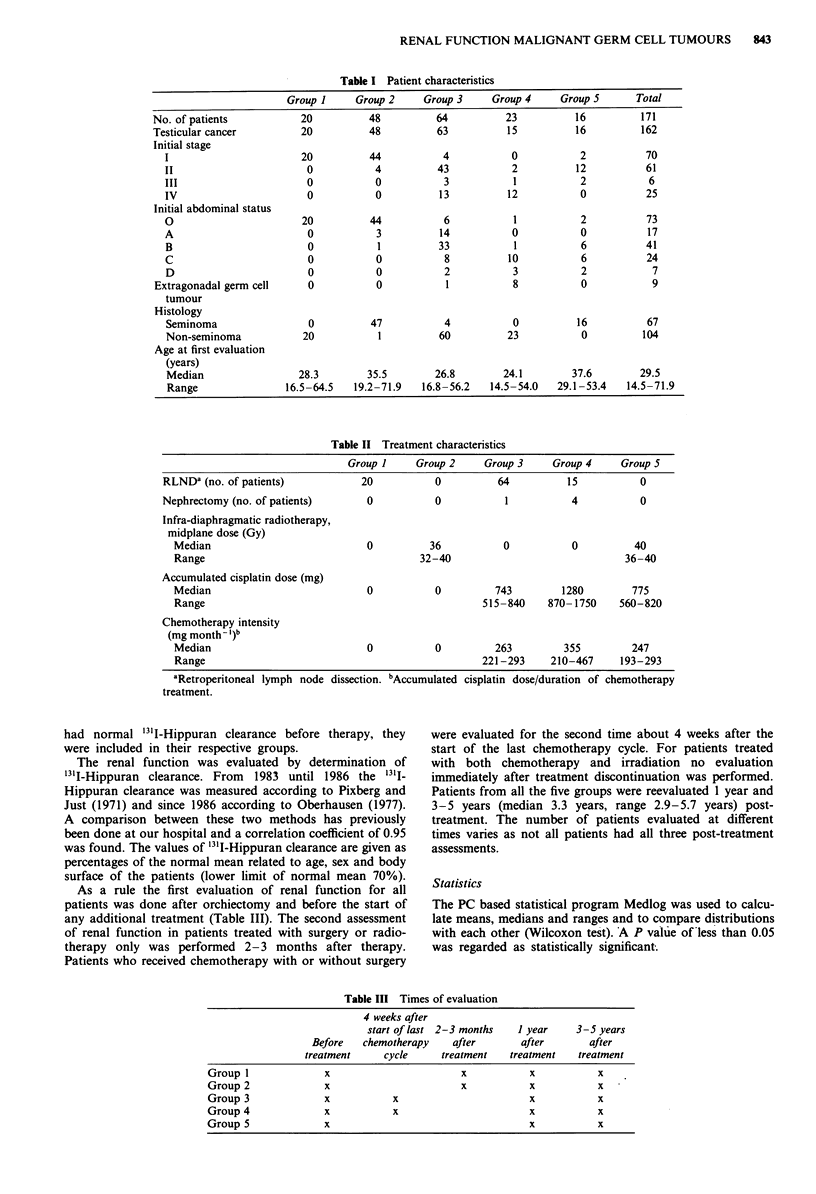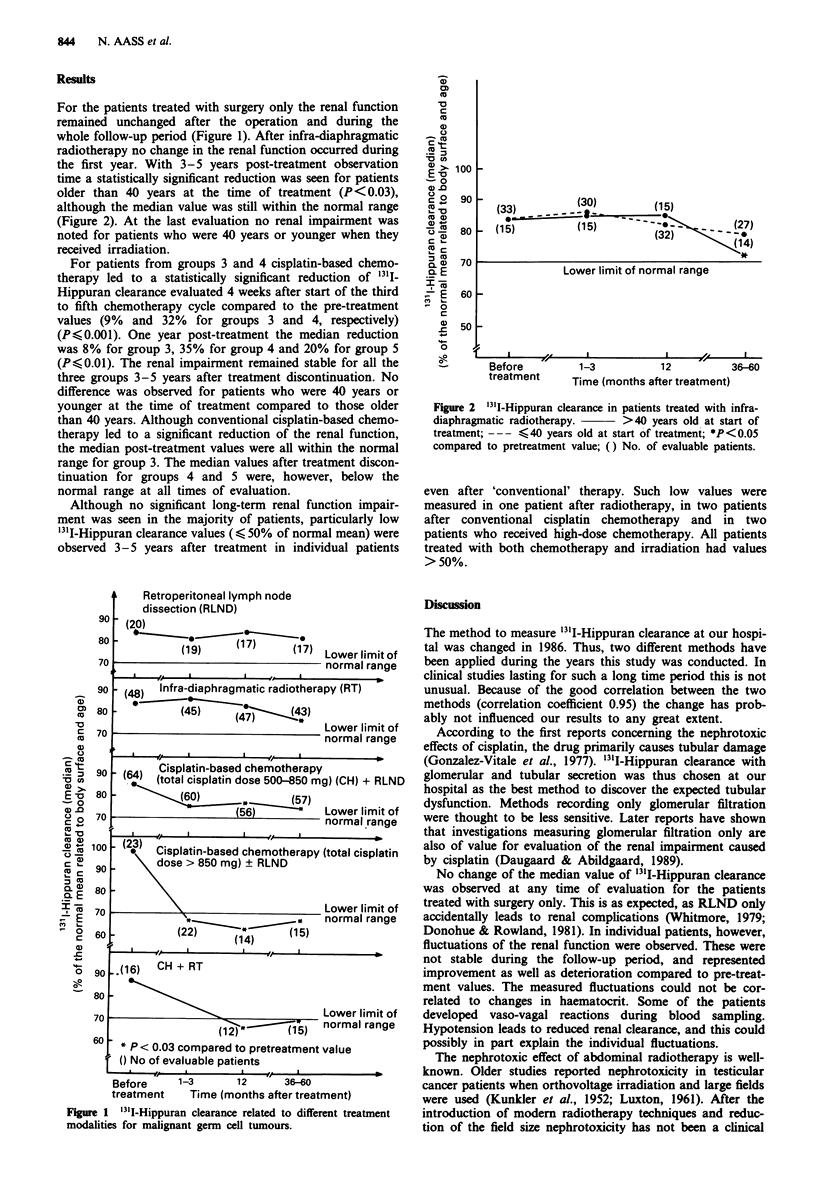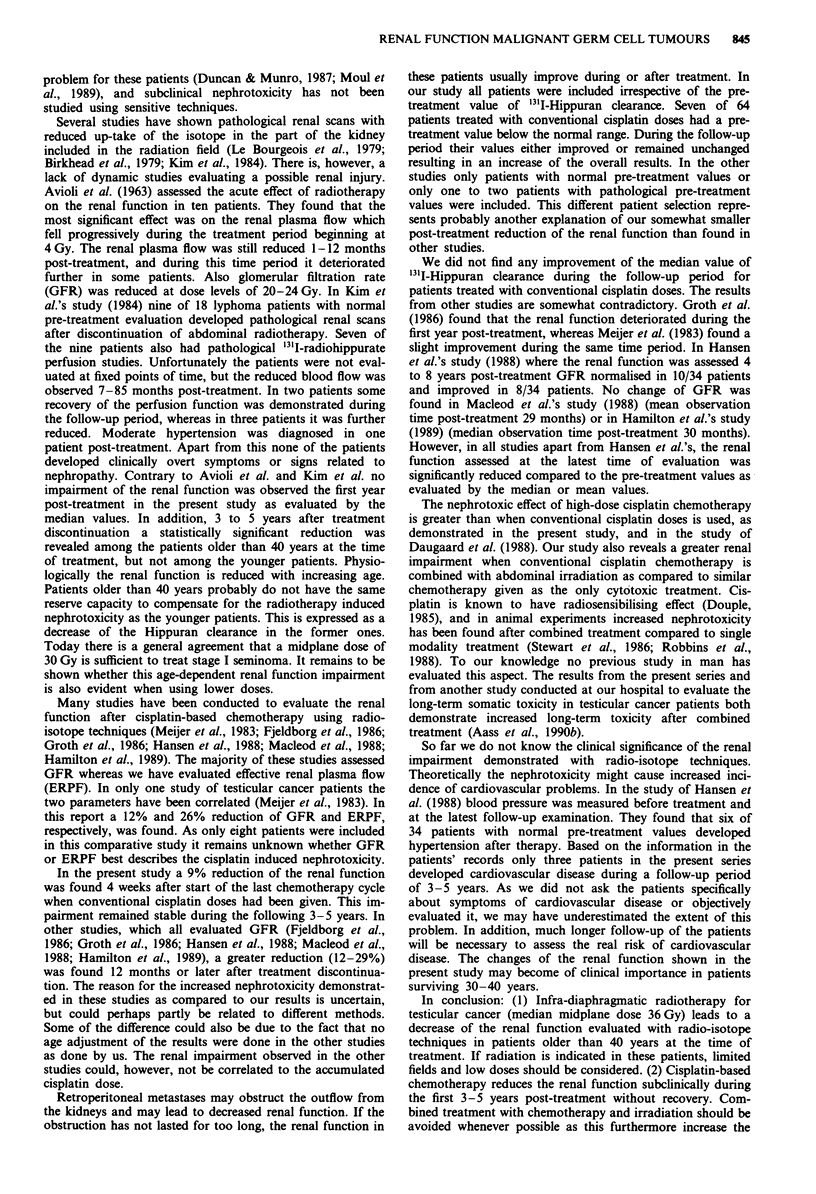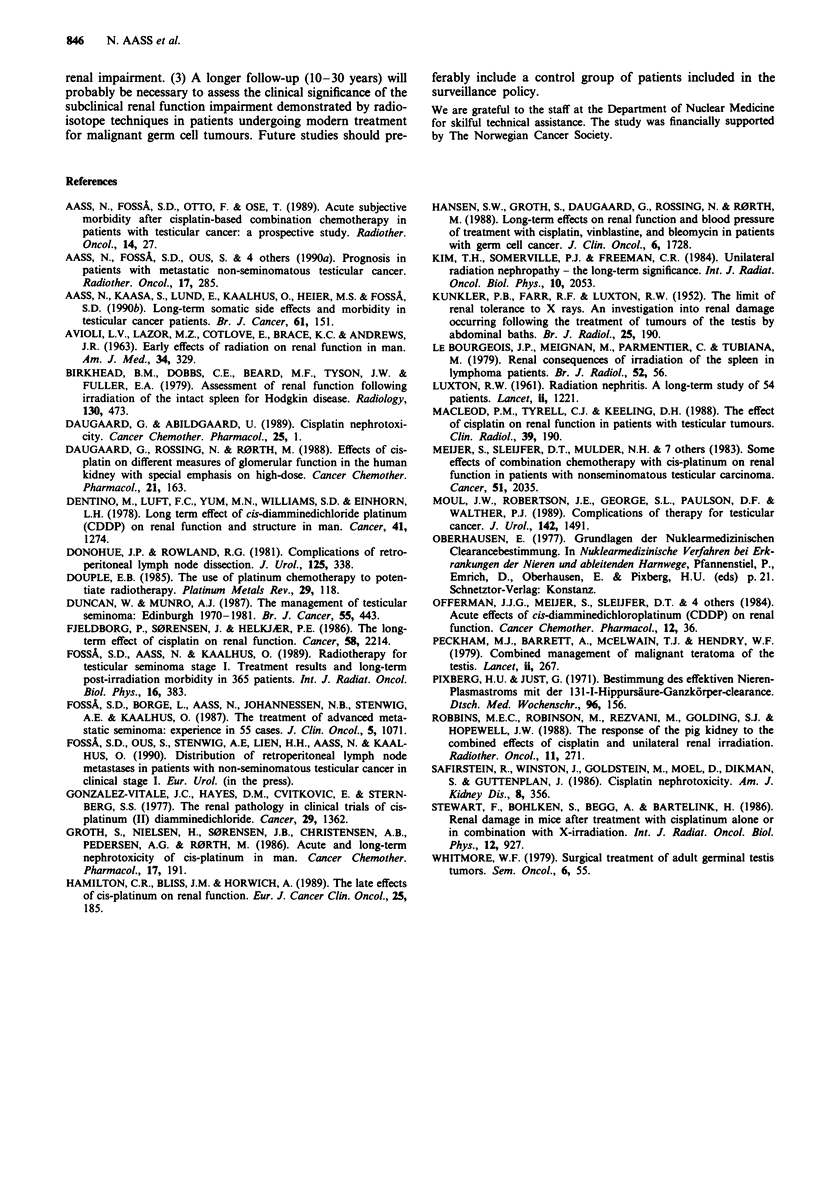# Renal function related to different treatment modalities for malignant germ cell tumours.

**DOI:** 10.1038/bjc.1990.391

**Published:** 1990-11

**Authors:** N. Aass, S. D. Fosså, M. Aas, M. W. Lindegaard

**Affiliations:** Department of Medical Oncology and Radiotherapy, Norwegian Radium Hospital, Oslo.

## Abstract

The renal function was evaluated with 131I-Hippuran clearance in 171 patients with malignant germ cell tumours. Assessments were performed before treatment and at three fixed times afterwards within 5 years. The patients were treated with surgery only (20 patients), infra-diaphragmatic radiotherapy only (median midplane dose 36 Gy) (48 patients), cisplatin-based chemotherapy (total cisplatin dose 500-850 mg) plus surgery (64 patients), cisplatin-based chemotherapy (total cisplatin dose greater than 850 mg) with or without surgery (23 patients) or cisplatin-based chemotherapy (total cisplatin dose 500-850 mg) plus infra-diaphragmatic radiotherapy (16 patients). No renal impairment was observed for patients treated with surgery only. In patients who received radiotherapy no change of the renal function occurred during the first year post-treatment. Three to five years after treatment discontinuation a statistically significant reduction within the normal range was observed in patients who were greater than 40 years at the time of irradiation. Cisplatin-based chemotherapy led to a statistically significant irreversible renal impairment for all the three groups. The greatest reduction was seen in patients who received the highest total cisplatin dose or who were treated with irradiation in addition to chemotherapy. The clinical significance of the observed nephrotoxicity is still unknown.


Br. J. Cancer (1990), 62, 842-846 © Macmillan Press Ltd., 1990

### Renal function related to different treatment modalities for malignant germ cell tumours

N. Aass[1], S.D. Fosså[1], M. Aas[2] & M.W. Lindegaard[2]

[1]*Department of Medical Oncology and Radiotherapy and* [2]*Department of Nuclear Medicine, The Norwegian Radium Hospital, Montebello, 0310 Oslo 3, Norway.*

**Summary** The renal function was evaluated with $^{131}$I-Hippuran clearance in 171 patients with malignant germ cell tumours. Assessments were performed before treatment and at three fixed times afterwards within 5 years. The patients were treated with surgery only (20 patients), infra-diaphragmatic radiotherapy only (median midplane dose 36 Gy) (48 patients), cisplatin-based chemotherapy (total cisplatin dose 500–850 mg) plus surgery (64 patients), cisplatin-based chemotherapy (total cisplatin dose > 850 mg) with or without surgery (23 patients) or cisplatin-based chemotherapy (total cisplatin dose 500–850 mg) plus infra-diaphragmatic radiotherapy (16 patients). No renal impairment was observed for patients treated with surgery only. In patients who received radiotherapy no change of the renal function occurred during the first year post-treatment. Three to five years after treatment discontinuation a statistically significant reduction within the normal range was observed in patients who were > 40 years at the time of irradiation. Cisplatin-based chemotherapy led to a statistically significant irreversible renal impairment for all the three groups. The greatest reduction was seen in patients who received the highest total cisplatin dose or who were treated with irradiation in addition to chemotherapy. The clinical significance of the observed nephrotoxicity is still unknown.

There is a lack of comparative studies regarding nephrotoxicity after different treatment modalities for testicular cancer. Renal toxicity is a well-known side effect after cisplatin-based combination chemotherapy (Dentino *et al.*, 1978; Offerman *et al.*, 1984; Safirstein *et al.*, 1986), but less attention has been paid to the possible renal impairment after high-voltage abdominal radiotherapy and the combination of chemotherapy and irradiation.

The aim of the present prospective study was to evaluate the renal function after retroperitoneal lymph node dissection, cisplatin-based chemotherapy, abdominal radiotherapy, and after combined treatment with chemotherapy and irradiation. Both the acute and the long-term results were assessed.

### Patients and methods

A total of 171 patients with malignant germ cell tumours (testicular 162, extragonadal nine) were included in the study (Table I). These patients represented a consecutive series of patients treated at The Norwegian Radium Hospital during two periods (January 1983 to December 1984 and July 1985 to October 1986). Staging was performed according to the Royal Marsden Staging System (Peckham *et al.*, 1979). The principles of primary treatment were as follows.

### *Seminoma*

Clinical stage I, IIa/b: infra-diaphragmatic radiotherapy 36–40 Gy (Fosså *et al.*, 1989).

Clinical stage ≥ IIc/extragonadal germ cell tumours: cisplatin-based combination chemotherapy (CVB: cisplatin 20 mg $m^{-2}$ days 1–5, vinblastine 0.10–0.15 mg $kg^{-1}$ day 1–2, bleomycin 30 mg days 2, 5, 15 (max. cumulative dose 300 mg)) followed by radiotherapy/surgery to initial tumour-bearing regions (Fosså *et al.*, 1987). From 1983 vinblastine was gradually replaced by VP-16 (500 mg $m^{-2}$ per cycle) (Aass *et al.*, 1989).

### *Non-seminoma*

Clinical stage I, IIa: retroperitoneal lymph node dissection (RLND) followed by three or four cycles of cisplatin-based chemotherapy in case of metastases (Fosså *et al.*, 1990).

Clinical stage ≥ IIb: cisplatin-based chemotherapy followed by surgery of residual masses within initial tumour-bearing areas (Aass *et al.*, 1990*a*). High-risk patients, as defined previously (Aass *et al.*, 1990*a*), were treated with a so-called high-dose cisplatin regimen (BEP60: cisplatin 60 mg $m^{-2}$ days 1–3, VP-16 120 mg $m^{-2}$ days 1–3, bleomycin 30 mg days 1, 5, 15 (max. cumulative dose 300 mg)).

### *Procedure*

The patients were divided into five different groups with regard to their overall treatment (Table II). Group 1: unilateral retroperitoneal lymph node dissection (RLND) (20 patients). Group 2: infra-diaphragmatic radiotherapy (48 patients). Group 3: cisplatin-based combination chemotherapy (total cisplatin dose 500–850 mg) + RLND (64 patients). Group 4: cisplatin-based combination chemotherapy (total cisplatin dose > 850 mg) ± RLND (23 patients). Group 5: cisplatin-based combination chemotherapy (total cisplatin dose 500–850 mg) + infra-diaphragmatic radiotherapy (16 patients).

The series includes ten patients treated for relapse by salvage chemotherapy. All patients in group 4 received at least one cycle with high-dose cisplatin chemotherapy (one cycle to one patient; two cycles to four patients; three cycles to nine patients; four cycles to nine patients). The other chemotherapy courses for this group and all courses for groups 3 and 5 were given with conventional cisplatin doses, i.e. 100 mg cisplatin $m^{-2}$ per cycle. The total number of chemotherapy cycles for patients in groups 3–5 were between three and eight cycles (most often four cycles).

Radiotherapy was given with linear accelerators (5–6 MV). Two opposed fields were given, one anterior and one posterior. The daily dose was 2 Gy (five fractions per week). One field was treated each day. Patients with stage I disease received a total dose of 36 Gy, whereas patients with metastatic disease received 40 Gy.

All patients in group 2 received treatment to a so-called L-field including the paraaortic and iliac regions. Patients in group 5 received either L-field irradiation or paraaortic radiotherapy. If more than one third of the renal tissue was included in the target volume, renal shielding was performed after 20 Gy.

Three patients had suffered from urolithiasis before treatment for the testicular cancer. In one of these patients partial nephrectomy had been performed. As all these three patients

Correspondence: N. Aass.
Received 22 March 1990; and in revised form 31 May 1990.

**Table I** Patient characteristics

| | *Group 1* | *Group 2* | *Group 3* | *Group 4* | *Group 5* | *Total* |
|---|---|---|---|---|---|---|
| No. of patients | 20 | 48 | 64 | 23 | 16 | 171 |
| Testicular cancer | 20 | 48 | 63 | 15 | 16 | 162 |
| Initial stage | | | | | | |
| I | 20 | 44 | 4 | 0 | 2 | 70 |
| II | 0 | 4 | 43 | 2 | 12 | 61 |
| III | 0 | 0 | 3 | 1 | 2 | 6 |
| IV | 0 | 0 | 13 | 12 | 0 | 25 |
| Initial abdominal status | | | | | | |
| O | 20 | 44 | 6 | 1 | 2 | 73 |
| A | 0 | 3 | 14 | 0 | 0 | 17 |
| B | 0 | 1 | 33 | 1 | 6 | 41 |
| C | 0 | 0 | 8 | 10 | 6 | 24 |
| D | 0 | 0 | 2 | 3 | 2 | 7 |
| Extragonadal germ cell tumour | 0 | 0 | 1 | 8 | 0 | 9 |
| Histology | | | | | | |
| Seminoma | 0 | 47 | 4 | 0 | 16 | 67 |
| Non-seminoma | 20 | 1 | 60 | 23 | 0 | 104 |
| Age at first evaluation (years) | | | | | | |
| Median | 28.3 | 35.5 | 26.8 | 24.1 | 37.6 | 29.5 |
| Range | 16.5–64.5 | 19.2–71.9 | 16.8–56.2 | 14.5–54.0 | 29.1–53.4 | 14.5–71.9 |

**Table II** Treatment characteristics

| | *Group 1* | *Group 2* | *Group 3* | *Group 4* | *Group 5* |
|---|---|---|---|---|---|
| RLND[a] (no. of patients) | 20 | 0 | 64 | 15 | 0 |
| Nephrectomy (no. of patients) | 0 | 0 | 1 | 4 | 0 |
| Infra-diaphragmatic radiotherapy, midplane dose (Gy) | | | | | |
| Median | 0 | 36 | 0 | 0 | 40 |
| Range | | 32–40 | | | 36–40 |
| Accumulated cisplatin dose (mg) | | | | | |
| Median | 0 | 0 | 743 | 1280 | 775 |
| Range | | | 515–840 | 870–1750 | 560–820 |
| Chemotherapy intensity (mg month$^{-1}$)[b] | | | | | |
| Median | 0 | 0 | 263 | 355 | 247 |
| Range | | | 221–293 | 210–467 | 193–293 |

[a]Retroperitoneal lymph node dissection. [b]Accumulated cisplatin dose/duration of chemotherapy treatment.

had normal $^{131}$I-Hippuran clearance before therapy, they were included in their respective groups.

The renal function was evaluated by determination of $^{131}$I-Hippuran clearance. From 1983 until 1986 the $^{131}$I-Hippuran clearance was measured according to Pixberg and Just (1971) and since 1986 according to Oberhausen (1977). A comparison between these two methods has previously been done at our hospital and a correlation coefficient of 0.95 was found. The values of $^{131}$I-Hippuran clearance are given as percentages of the normal mean related to age, sex and body surface of the patients (lower limit of normal mean 70%).

As a rule the first evaluation of renal function for all patients was done after orchiectomy and before the start of any additional treatment (Table III). The second assessment of renal function in patients treated with surgery or radiotherapy only was performed 2–3 months after therapy. Patients who received chemotherapy with or without surgery were evaluated for the second time about 4 weeks after the start of the last chemotherapy cycle. For patients treated with both chemotherapy and irradiation no evaluation immediately after treatment discontinuation was performed. Patients from all the five groups were reevaluated 1 year and 3–5 years (median 3.3 years, range 2.9–5.7 years) post-treatment. The number of patients evaluated at different times varies as not all patients had all three post-treatment assessments.

### *Statistics*

The PC based statistical program Medlog was used to calculate means, medians and ranges and to compare distributions with each other (Wilcoxon test). A $P$ value of less than 0.05 was regarded as statistically significant.

**Table III** Times of evaluation

| | *Before treatment* | *4 weeks after start of last chemotherapy cycle* | *2–3 months after treatment* | *1 year after treatment* | *3–5 years after treatment* |
|---|---|---|---|---|---|
| Group 1 | x | | x | x | x |
| Group 2 | x | | x | x | x |
| Group 3 | x | x | | x | x |
| Group 4 | x | x | | x | x |
| Group 5 | x | | | x | x |

### Results

For the patients treated with surgery only the renal function remained unchanged after the operation and during the whole follow-up period (Figure 1). After infra-diaphragmatic radiotherapy no change in the renal function occurred during the first year. With 3–5 years post-treatment observation time a statistically significant reduction was seen for patients older than 40 years at the time of treatment ($P<0.03$), although the median value was still within the normal range (Figure 2). At the last evaluation no renal impairment was noted for patients who were 40 years or younger when they received irradiation.

For patients from groups 3 and 4 cisplatin-based chemotherapy led to a statistically significant reduction of $^{131}$I-Hippuran clearance evaluated 4 weeks after start of the third to fifth chemotherapy cycle compared to the pre-treatment values (9% and 32% for groups 3 and 4, respectively) ($P\leqslant 0.001$). One year post-treatment the median reduction was 8% for group 3, 35% for group 4 and 20% for group 5 ($P\leqslant 0.01$). The renal impairment remained stable for all the three groups 3–5 years after treatment discontinuation. No difference was observed for patients who were 40 years or younger at the time of treatment compared to those older than 40 years. Although conventional cisplatin-based chemotherapy led to a significant reduction of the renal function, the median post-treatment values were all within the normal range for group 3. The median values after treatment discontinuation for groups 4 and 5 were, however, below the normal range at all times of evaluation.

Although no significant long-term renal function impairment was seen in the majority of patients, particularly low $^{131}$I-Hippuran clearance values ($\leqslant 50\%$ of normal mean) were observed 3–5 years after treatment in individual patients even after 'conventional' therapy. Such low values were measured in one patient after radiotherapy, in two patients after conventional cisplatin chemotherapy and in two patients who received high-dose chemotherapy. All patients treated with both chemotherapy and irradiation had values $>50\%$.

**Figure 2** $^{131}$I-Hippuran clearance in patients treated with infra-diaphragmatic radiotherapy. ——— >40 years old at start of treatment; - - - ≤40 years old at start of treatment; *$P<0.05$ compared to pretreatment value; ( ) No. of evaluable patients.

**Figure 1** $^{131}$I-Hippuran clearance related to different treatment modalities for malignant germ cell tumours.

### Discussion

The method to measure $^{131}$I-Hippuran clearance at our hospital was changed in 1986. Thus, two different methods have been applied during the years this study was conducted. In clinical studies lasting for such a long time period this is not unusual. Because of the good correlation between the two methods (correlation coefficient 0.95) the change has probably not influenced our results to any great extent.

According to the first reports concerning the nephrotoxic effects of cisplatin, the drug primarily causes tubular damage (Gonzalez-Vitale *et al.*, 1977). $^{131}$I-Hippuran clearance with glomerular and tubular secretion was thus chosen at our hospital as the best method to discover the expected tubular dysfunction. Methods recording only glomerular filtration were thought to be less sensitive. Later reports have shown that investigations measuring glomerular filtration only are also of value for evaluation of the renal impairment caused by cisplatin (Daugaard & Abildgaard, 1989).

No change of the median value of $^{131}$I-Hippuran clearance was observed at any time of evaluation for the patients treated with surgery only. This is as expected, as RLND only accidentally leads to renal complications (Whitmore, 1979; Donohue & Rowland, 1981). In individual patients, however, fluctuations of the renal function were observed. These were not stable during the follow-up period, and represented improvement as well as deterioration compared to pre-treatment values. The measured fluctuations could not be correlated to changes in haematocrit. Some of the patients developed vaso-vagal reactions during blood sampling. Hypotension leads to reduced renal clearance, and this could possibly in part explain the individual fluctuations.

The nephrotoxic effect of abdominal radiotherapy is well-known. Older studies reported nephrotoxicity in testicular cancer patients when orthovoltage irradiation and large fields were used (Kunkler *et al.*, 1952; Luxton, 1961). After the introduction of modern radiotherapy techniques and reduction of the field size nephrotoxicity has not been a clinical problem for these patients (Duncan & Munro, 1987; Moul *et al.*, 1989), and subclinical nephrotoxicity has not been studied using sensitive techniques.

Several studies have shown pathological renal scans with reduced up-take of the isotope in the part of the kidney included in the radiation field (Le Bourgeois *et al.*, 1979; Birkhead *et al.*, 1979; Kim *et al.*, 1984). There is, however, a lack of dynamic studies evaluating a possible renal injury. Avioli *et al.* (1963) assessed the acute effect of radiotherapy on the renal function in ten patients. They found that the most significant effect was on the renal plasma flow which fell progressively during the treatment period beginning at 4 Gy. The renal plasma flow was still reduced 1–12 months post-treatment, and during this time period it deteriorated further in some patients. Also glomerular filtration rate (GFR) was reduced at dose levels of 20–24 Gy. In Kim *et al.*'s study (1984) nine of 18 lyphoma patients with normal pre-treatment evaluation developed pathological renal scans after discontinuation of abdominal radiotherapy. Seven of the nine patients also had pathological $^{131}$I-radiohippurate perfusion studies. Unfortunately the patients were not evaluated at fixed points of time, but the reduced blood flow was observed 7–85 months post-treatment. In two patients some recovery of the perfusion function was demonstrated during the follow-up period, whereas in three patients it was further reduced. Moderate hypertension was diagnosed in one patient post-treatment. Apart from this none of the patients developed clinically overt symptoms or signs related to nephropathy. Contrary to Avioli *et al.* and Kim *et al.* no impairment of the renal function was observed the first year post-treatment in the present study as evaluated by the median values. In addition, 3 to 5 years after treatment discontinuation a statistically significant reduction was revealed among the patients older than 40 years at the time of treatment, but not among the younger patients. Physiologically the renal function is reduced with increasing age. Patients older than 40 years probably do not have the same reserve capacity to compensate for the radiotherapy induced nephrotoxicity as the younger patients. This is expressed as a decrease of the Hippuran clearance in the former ones. Today there is a general agreement that a midplane dose of 30 Gy is sufficient to treat stage I seminoma. It remains to be shown whether this age-dependent renal function impairment is also evident when using lower doses.

Many studies have been conducted to evaluate the renal function after cisplatin-based chemotherapy using radio-isotope techniques (Meijer *et al.*, 1983; Fjeldborg *et al.*, 1986; Groth *et al.*, 1986; Hansen *et al.*, 1988; Macleod *et al.*, 1988; Hamilton *et al.*, 1989). The majority of these studies assessed GFR whereas we have evaluated effective renal plasma flow (ERPF). In only one study of testicular cancer patients the two parameters have been correlated (Meijer *et al.*, 1983). In this report a 12% and 26% reduction of GFR and ERPF, respectively, was found. As only eight patients were included in this comparative study it remains unknown whether GFR or ERPF best describes the cisplatin induced nephrotoxicity.

In the present study a 9% reduction of the renal function was found 4 weeks after start of the last chemotherapy cycle when conventional cisplatin doses had been given. This impairment remained stable during the following 3–5 years. In other studies, which all evaluated GFR (Fjeldborg *et al.*, 1986; Groth *et al.*, 1986; Hansen *et al.*, 1988; Macleod *et al.*, 1988; Hamilton *et al.*, 1989), a greater reduction (12–29%) was found 12 months or later after treatment discontinuation. The reason for the increased nephrotoxicity demonstrated in these studies as compared to our results is uncertain, but could perhaps partly be related to different methods. Some of the difference could also be due to the fact that no age adjustment of the results were done in the other studies as done by us. The renal impairment observed in the other studies could, however, not be correlated to the accumulated cisplatin dose.

Retroperitoneal metastases may obstruct the outflow from the kidneys and may lead to decreased renal function. If the obstruction has not lasted for too long, the renal function in these patients usually improve during or after treatment. In our study all patients were included irrespective of the pre-treatment value of $^{131}$I-Hippuran clearance. Seven of 64 patients treated with conventional cisplatin doses had a pre-treatment value below the normal range. During the follow-up period their values either improved or remained unchanged resulting in an increase of the overall results. In the other studies only patients with normal pre-treatment values or only one to two patients with pathological pre-treatment values were included. This different patient selection represents probably another explanation of our somewhat smaller post-treatment reduction of the renal function than found in other studies.

We did not find any improvement of the median value of $^{131}$I-Hippuran clearance during the follow-up period for patients treated with conventional cisplatin doses. The results from other studies are somewhat contradictory. Groth *et al.* (1986) found that the renal function deteriorated during the first year post-treatment, whereas Meijer *et al.* (1983) found a slight improvement during the same time period. In Hansen *et al.*'s study (1988) where the renal function was assessed 4 to 8 years post-treatment GFR normalised in 10/34 patients and improved in 8/34 patients. No change of GFR was found in Macleod *et al.*'s study (1988) (mean observation time post-treatment 29 months) or in Hamilton *et al.*'s study (1989) (median observation time post-treatment 30 months). However, in all studies apart from Hansen *et al.*'s, the renal function assessed at the latest time of evaluation was significantly reduced compared to the pre-treatment values as evaluated by the median or mean values.

The nephrotoxic effect of high-dose cisplatin chemotherapy is greater than when conventional cisplatin doses is used, as demonstrated in the present study, and in the study of Daugaard *et al.* (1988). Our study also reveals a greater renal impairment when conventional cisplatin chemotherapy is combined with abdominal irradiation as compared to similar chemotherapy given as the only cytotoxic treatment. Cisplatin is known to have radiosensibilising effect (Douple, 1985), and in animal experiments increased nephrotoxicity has been found after combined treatment compared to single modality treatment (Stewart *et al.*, 1986; Robbins *et al.*, 1988). To our knowledge no previous study in man has evaluated this aspect. The results from the present series and from another study conducted at our hospital to evaluate the long-term somatic toxicity in testicular cancer patients both demonstrate increased long-term toxicity after combined treatment (Aass *et al.*, 1990*b*).

So far we do not know the clinical significance of the renal impairment demonstrated with radio-isotope techniques. Theoretically the nephrotoxicity might cause increased incidence of cardiovascular problems. In the study of Hansen *et al.* (1988) blood pressure was measured before treatment and at the latest follow-up examination. They found that six of 34 patients with normal pre-treatment values developed hypertension after therapy. Based on the information in the patients' records only three patients in the present series developed cardiovascular disease during a follow-up period of 3–5 years. As we did not ask the patients specifically about symptoms of cardiovascular disease or objectively evaluated it, we may have underestimated the extent of this problem. In addition, much longer follow-up of the patients will be necessary to assess the real risk of cardiovascular disease. The changes of the renal function shown in the present study may become of clinical importance in patients surviving 30–40 years.

In conclusion: (1) Infra-diaphragmatic radiotherapy for testicular cancer (median midplane dose 36 Gy) leads to a decrease of the renal function evaluated with radio-isotope techniques in patients older than 40 years at the time of treatment. If radiation is indicated in these patients, limited fields and low doses should be considered. (2) Cisplatin-based chemotherapy reduces the renal function subclinically during the first 3–5 years post-treatment without recovery. Combined treatment with chemotherapy and irradiation should be avoided whenever possible as this furthermore increase the renal impairment. (3) A longer follow-up (10–30 years) will probably be necessary to assess the clinical significance of the subclinical renal function impairment demonstrated by radioisotope techniques in patients undergoing modern treatment for malignant germ cell tumours. Future studies should preferably include a control group of patients included in the surveillance policy.

We are grateful to the staff at the Department of Nuclear Medicine for skilful technical assistance. The study was financially supported by The Norwegian Cancer Society.

### References

AASS, N., FOSSÅ, S.D., OTTO, F. & OSE, T. (1989). Acute subjective morbidity after cisplatin-based combination chemotherapy in patients with testicular cancer: a prospective study. *Radiother. Oncol.*, **14**, 27.

AASS, N., FOSSÅ, S.D., OUS, S. & 4 others (1990*a*). Prognosis in patients with metastatic non-seminomatous testicular cancer. *Radiother. Oncol.*, **17**, 285.

AASS, N., KAASA, S., LUND, E., KAALHUS, O., HEIER, M.S. & FOSSÅ, S.D. (1990*b*). Long-term somatic side effects and morbidity in testicular cancer patients. *Br. J. Cancer*, **61**, 151.

AVIOLI, L.V., LAZOR, M.Z., COTLOVE, E., BRACE, K.C. & ANDREWS, J.R. (1963). Early effects of radiation on renal function in man. *Am. J. Med.*, **34**, 329.

BIRKHEAD, B.M., DOBBS, C.E., BEARD, M.F., TYSON, J.W. & FULLER, E.A. (1979). Assessment of renal function following irradiation of the intact spleen for Hodgkin disease. *Radiology*, **130**, 473.

DAUGAARD, G. & ABILDGAARD, U. (1989). Cisplatin nephrotoxicity. *Cancer Chemother. Pharmacol.*, **25**, 1.

DAUGAARD, G., ROSSING, N. & RØRTH, M. (1988). Effects of cisplatin on different measures of glomerular function in the human kidney with special emphasis on high-dose. *Cancer Chemother. Pharmacol.*, **21**, 163.

DENTINO, M., LUFT, F.C., YUM, M.N., WILLIAMS, S.D. & EINHORN, L.H. (1978). Long term effect of *cis*-diamminedichloride platinum (CDDP) on renal function and structure in man. *Cancer*, **41**, 1274.

DONOHUE, J.P. & ROWLAND, R.G. (1981). Complications of retroperitoneal lymph node dissection. *J. Urol.*, **125**, 338.

DOUPLE, E.B. (1985). The use of platinum chemotherapy to potentiate radiotherapy. *Platinum Metals Rev.*, **29**, 118.

DUNCAN, W. & MUNRO, A.J. (1987). The management of testicular seminoma: Edinburgh 1970–1981. *Br. J. Cancer*, **55**, 443.

FJELDBORG, P., SØRENSEN, J. & HELKJÆR, P.E. (1986). The long-term effect of cisplatin on renal function. *Cancer*, **58**, 2214.

FOSSÅ, S.D., AASS, N. & KAALHUS, O. (1989). Radiotherapy for testicular seminoma stage I. Treatment results and long-term post-irradiation morbidity in 365 patients. *Int. J. Radiat. Oncol. Biol. Phys.*, **16**, 383.

FOSSÅ, S.D., BORGE, L., AASS, N., JOHANNESSEN, N.B., STENWIG, A.E. & KAALHUS, O. (1987). The treatment of advanced metastatic seminoma: experience in 55 cases. *J. Clin. Oncol.*, **5**, 1071.

FOSSÅ, S.D., OUS, S., STENWIG, A.E, LIEN, H.H., AASS, N. & KAALHUS, O. (1990). Distribution of retroperitoneal lymph node metastases in patients with non-seminomatous testicular cancer in clinical stage I. *Eur. Urol.* (in the press).

GONZALEZ-VITALE, J.C., HAYES, D.M., CVITKOVIC, E. & STERNBERG, S.S. (1977). The renal pathology in clinical trials of cisplatinum (II) diamminedichloride. *Cancer*, **29**, 1362.

GROTH, S., NIELSEN, H., SØRENSEN, J.B., CHRISTENSEN, A.B., PEDERSEN, A.G. & RØRTH, M. (1986). Acute and long-term nephrotoxicity of cis-platinum in man. *Cancer Chemother. Pharmacol.*, **17**, 191.

HAMILTON, C.R., BLISS, J.M. & HORWICH, A. (1989). The late effects of cis-platinum on renal function. *Eur. J. Cancer Clin. Oncol.*, **25**, 185.

HANSEN, S.W., GROTH, S., DAUGAARD, G., ROSSING, N. & RØRTH, M. (1988). Long-term effects on renal function and blood pressure of treatment with cisplatin, vinblastine, and bleomycin in patients with germ cell cancer. *J. Clin. Oncol.*, **6**, 1728.

KIM, T.H., SOMERVILLE, P.J. & FREEMAN, C.R. (1984). Unilateral radiation nephropathy – the long-term significance. *Int. J. Radiat. Oncol. Biol. Phys.*, **10**, 2053.

KUNKLER, P.B., FARR, R.F. & LUXTON, R.W. (1952). The limit of renal tolerance to X rays. An investigation into renal damage occurring following the treatment of tumours of the testis by abdominal baths. *Br. J. Radiol.*, **25**, 190.

LE BOURGEOIS, J.P., MEIGNAN, M., PARMENTIER, C. & TUBIANA, M. (1979). Renal consequences of irradiation of the spleen in lymphoma patients. *Br. J. Radiol.*, **52**, 56.

LUXTON, R.W. (1961). Radiation nephritis. A long-term study of 54 patients. *Lancet*, **ii**, 1221.

MACLEOD, P.M., TYRELL, C.J. & KEELING, D.H. (1988). The effect of cisplatin on renal function in patients with testicular tumours. *Clin. Radiol.*, **39**, 190.

MEIJER, S., SLEIJFER, D.T., MULDER, N.H. & 7 others (1983). Some effects of combination chemotherapy with cis-platinum on renal function in patients with nonseminomatous testicular carcinoma. *Cancer*, **51**, 2035.

MOUL, J.W., ROBERTSON, J.E., GEORGE, S.L., PAULSON, D.F. & WALTHER, P.J. (1989). Complications of therapy for testicular cancer. *J. Urol.*, **142**, 1491.

OBERHAUSEN, E. (1977). Grundlagen der Nuklearmedizinischen Clearancebestimmung. In *Nuklearmedizinische Verfahren bei Erkrankungen der Nieren und ableitenden Harnwege*, Pfannenstiel, P., Emrich, D., Oberhausen, E. & Pixberg, H.U. (eds) p. 21. Schnetztor-Verlag: Konstanz.

OFFERMAN, J.J.G., MEIJER, S., SLEIJFER, D.T. & 4 others (1984). Acute effects of *cis*-diamminedichloroplatinum (CDDP) on renal function. *Cancer Chemother. Pharmacol.*, **12**, 36.

PECKHAM, M.J., BARRETT, A., MCELWAIN, T.J. & HENDRY, W.F. (1979). Combined management of malignant teratoma of the testis. *Lancet*, **ii**, 267.

PIXBERG, H.U. & JUST, G. (1971). Bestimmung des effektiven Nieren-Plasmastroms mit der 131-I-Hippursäure-Ganzkörper-clearance. *Dtsch. Med. Wochenschr.*, **96**, 156.

ROBBINS, M.E.C., ROBINSON, M., REZVANI, M., GOLDING, S.J. & HOPEWELL, J.W. (1988). The response of the pig kidney to the combined effects of cisplatin and unilateral renal irradiation. *Radiother. Oncol.*, **11**, 271.

SAFIRSTEIN, R., WINSTON, J., GOLDSTEIN, M., MOEL, D., DIKMAN, S. & GUTTENPLAN, J. (1986). Cisplatin nephrotoxicity. *Am. J. Kidney Dis.*, **8**, 356.

STEWART, F., BOHLKEN, S., BEGG, A. & BARTELINK, H. (1986). Renal damage in mice after treatment with cisplatinum alone or in combination with X-irradiation. *Int. J. Radiat. Oncol. Biol. Phys.*, **12**, 927.

WHITMORE, W.F. (1979). Surgical treatment of adult germinal testis tumors. *Sem. Oncol.*, **6**, 55.